# Ultrasmall magnetic field-effect and sign reversal in transistors based on donor/acceptor systems

**DOI:** 10.3762/bjnano.8.112

**Published:** 2017-05-19

**Authors:** Thomas Reichert, Tobat P I Saragi

**Affiliations:** 1 Macromolecular Chemistry and Molecular Materials, Department of Mathematics and Science, Center for Interdisciplinary Nanostructure Science and Technology (CINSaT), University of Kassel, Heinrich-Plett-Straße 40, 34132 Kassel, Germany; 2 Accenture GmbH, Kaistraße 20, 40221 Düsseldorf, Germany

**Keywords:** donor/acceptor system, organic magnetoresistance, organic transistors, sign reversal, ultrasmall magnetic field-effects

## Abstract

We present magnetoresistive organic field-effect transistors featuring ultrasmall magnetic field-effects as well as a sign reversal. The employed material systems are coevaporated thin films with different compositions consisting of the electron donor 2,2',7,7'-tetrakis-(*N*,*N*-di-*p*-methylphenylamino)-9,9'-spirobifluorene (Spiro-TTB) and the electron acceptor 1,4,5,8,9,12-hexaazatriphenylene hexacarbonitrile (HAT-CN). Intermolecular charge transfer between Spiro-TTB and HAT-CN results in a high intrinsic charge carrier density in the coevaporated films. This enhances the probability of bipolaron formation, which is the process responsible for magnetoresistance effects in our system. Thereby even ultrasmall magnetic fields as low as 0.7 mT can influence the resistance of the charge transport channel. Moreover, the magnetoresistance is drastically influenced by the drain voltage, resulting in a sign reversal. An average *B*_0_ value of ≈2.1 mT is obtained for all mixing compositions, indicating that only one specific quasiparticle is responsible for the magnetoresistance effects. All magnetoresistance effects can be thoroughly clarified within the framework of the bipolaron model.

## Introduction

In recent years, the development of organic π-conjugated materials have perfectly meet the requirements for low-cost and flexible electronic devices and optoelectronic applications such as displays and lighting, which are already commercially available. To push organic electronics to the next level, the scientific community is shifting its activities towards the study of spin transport and spin phenomena in organic materials. Generally, three different topics are in the focus of this emerging research field, namely organic spin-valves [1–6], organic magnetoresistance [7–12] and spin-related effects in hybrid devices [13–15].

Research on organic spintronics have mostly used a diode as device structure (two-terminal device). In contrast, our tool in organic spintronics are field-effect transistors (three-terminal device), which gain new insight into spin transport phenomena in organic π-conjugated materials. In field-effect transistors, the sign of charge carriers is defined by the gate voltage, and the mobility and charge type can be determined independently [16]. The possibility of injecting holes or electrons or both into the conduction channel allows us to address the transport regime individually, depending on the applied drain and gate voltages. One interesting material system for transistors is the charge-transfer complex molecule, which this system has already shown magnetoresistive effects in organic diodes [17]. In a previous study we presented the first magnetoresistive effects in transistor structures based on a coevaporated (50:50) thin film materials system consisting of 2,2',7,7'-tetrakis-(*N*,*N*-di-*p*-methylphenylamino)-9,9'-spirobifluorene (Spiro-TTB) as donor and 1,4,5,8,9,12-hexaazatriphenylene hexacarbonitrile (HAT-CN) as acceptor [18]. While transistors based on the pure individual compounds are not influenced by external magnetic fields, their coevaporation allows the fabrication of highly magnetosensitive devices. With these findings, we were the first to establish donor–acceptor interactions as one powerful way to create magnetosensitive transistors but we could not yet cover all experimental aspects of this materials system.

**Figure 1:**
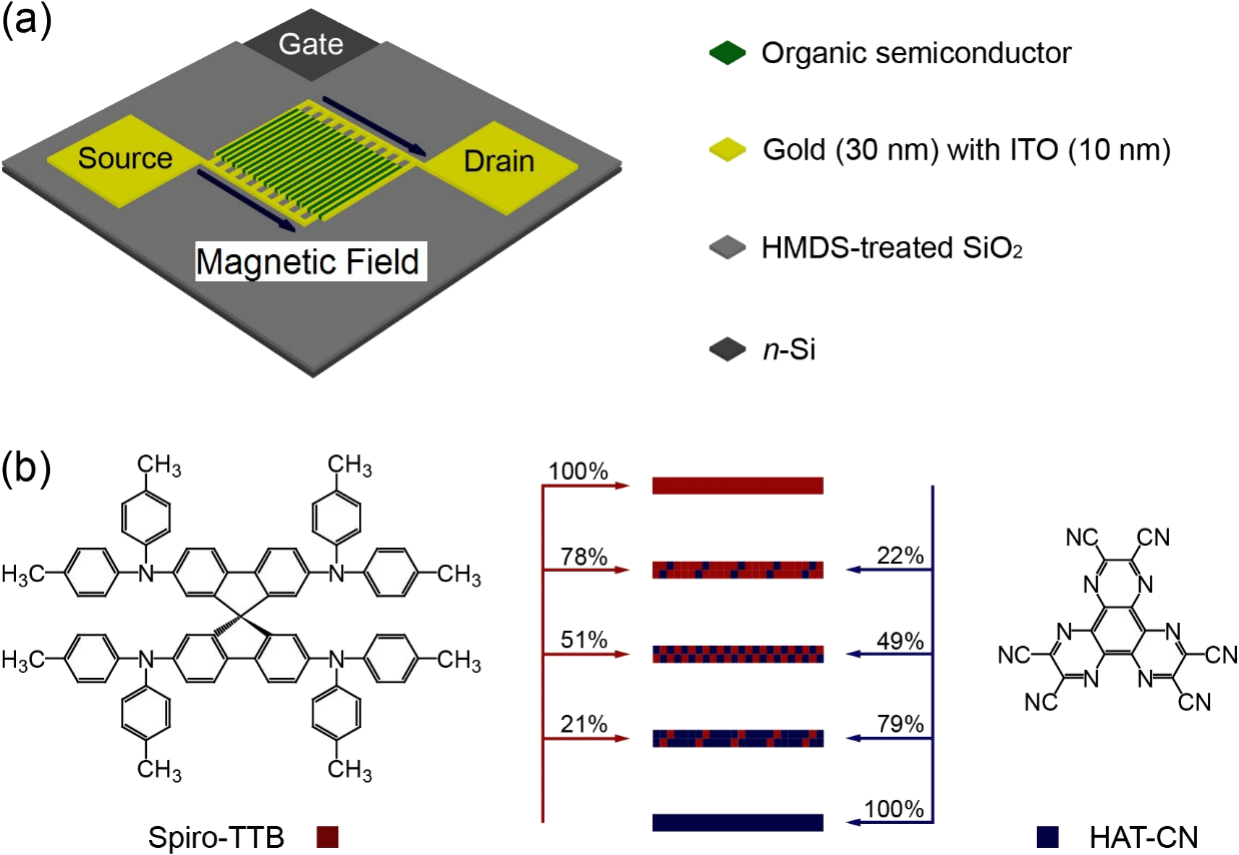
(a) Device layout of a bottom-contact organic field-effect transistor, showing *n*-Si as gate electrode, HMDS-treated SiO_2_ as gate dielectric, gold with ITO as source and drain electrodes and organic semiconductor. (b) The chemical structures of Spiro-TTB and HAT-CN, including mixing compositions of 78:22, 51:49 and 21:79 for Spiro-TTB/HAT-CN, respectively. For a better overview, the organic semiconductor thin film is only depicted in the region of the transistor channel.

In this paper, we will describe several important themes not featured in our previous paper. Further experimental investigations including different mixing compositions reveal a voltage-induced sign reversal and allow the first in-depth study of ultrasmall magnetic-field effects in transistor structures. In particular, the influence of the drain and the gate voltage will be described. We also present magnetoresistive effects at ultrasmall fields as low as 0.7 mT. Figure 1 shows the applied experimental scheme, the corresponding experimental parameters and the chemical structures of the active materials. The details of the experiments can be found in the experimental section of this paper.

## Results and Discussion

**Figure 2:**
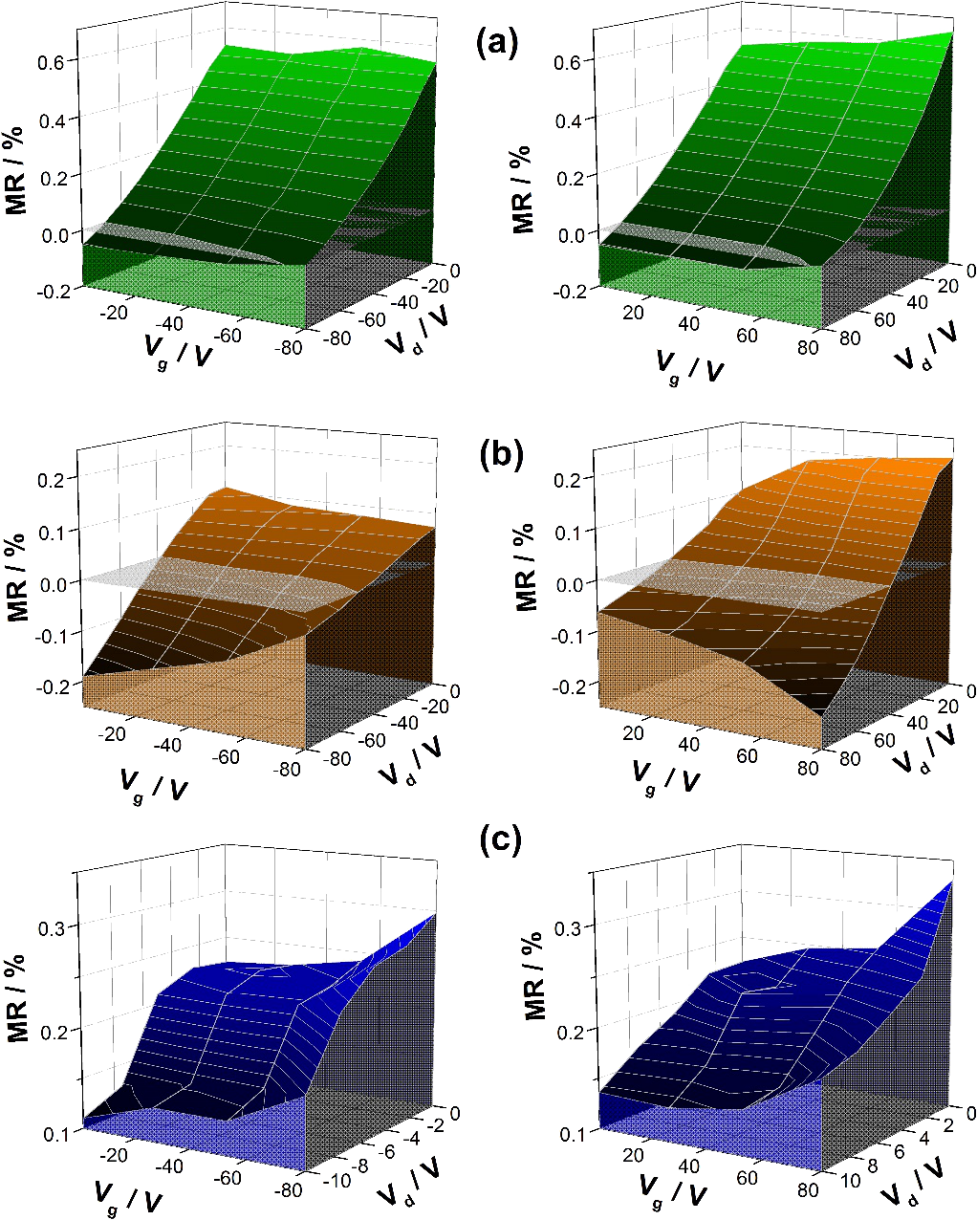
Voltage dependence (*V*_d_ and *V*_g_) of the magnetoresistance for compositions of (a) 51:49, (b) 78:22 and (c) 21:79. On the left side, *p*-channel conditions are shown, while *n*-channel conditions are depicted on the right side. In order to allow a continuous 3D representation of the explored magnetoresistance landscape, the Renka–Cline fit was used to interpolate between all experimental data points. The underlying experimental data is displayed in Supporting Information File 1, Figure S2 [19]. The inserted grey-shaded plane serves to illustrate the voltage-induced sign reversal. All measurements were carried out for *B* = 60 mT.

Coevaporating Spiro-TTB and HAT-CN results in mixed thin-film systems containing similar surface-morphologies as the corresponding “single” films suggesting an insignificant influence of the morphology on the magnetoresistive behaviour (see Supporting Information File 1, Figure S1). Instead, the magnetoresistance dependence on the source- and drain voltages as shown in Figure 2 for different mixing ratios. All compositions behave qualitatively equal for *p-*channel as well as for *n*-channel conditions. Thereby the gate voltage *V*_g_ does not significantly influence the magnetoresistance, but it strongly depends on the drain voltage *V*_d_. These trends consolidate the conclusions drawn from the electrical characterization, showing a relatively gate-independent transport behaviour [18]. Due to the charge transfer between the HOMO of Spiro-TTB (*E*_HOMO_ = −4.9 eV) and the LUMO of HAT-CN (*E*_LUMO_ = −5.1 eV), the intrinsically available number of holes and electrons is so large that the gate-induced charge carrier accumulation does not has a significant influence on the drain current *I*_d_ [18]. Therefore, *V*_d_ does not only dominate the charge transport but also the magnetotransport behaviour. For small *V*_d_ and for devices containing mixing ratios of 51:49 and 78:22 there is a positive magnetoresistance, which decreases with an increase of |*V*_d_| until reaching a sign reversal, as displayed in Figure 2a and 2b. Indications of the voltage-induced sign reversal can also be found in the data of 21:79 composition, as shown in Figure 2c. Unfortunately, it could not be detected experimentally because *V*_d_ values higher than 10 V result in current values above the upper limit of our measurement setup. However, the data also shows a reduction of the positive magnetoresistance with increasing of |*V*_d_|. This implies that the sign of magnetoresistance can be changed electrically in all Spiro-TTB/HAT-CN mixing ratios.

**Figure 3:**
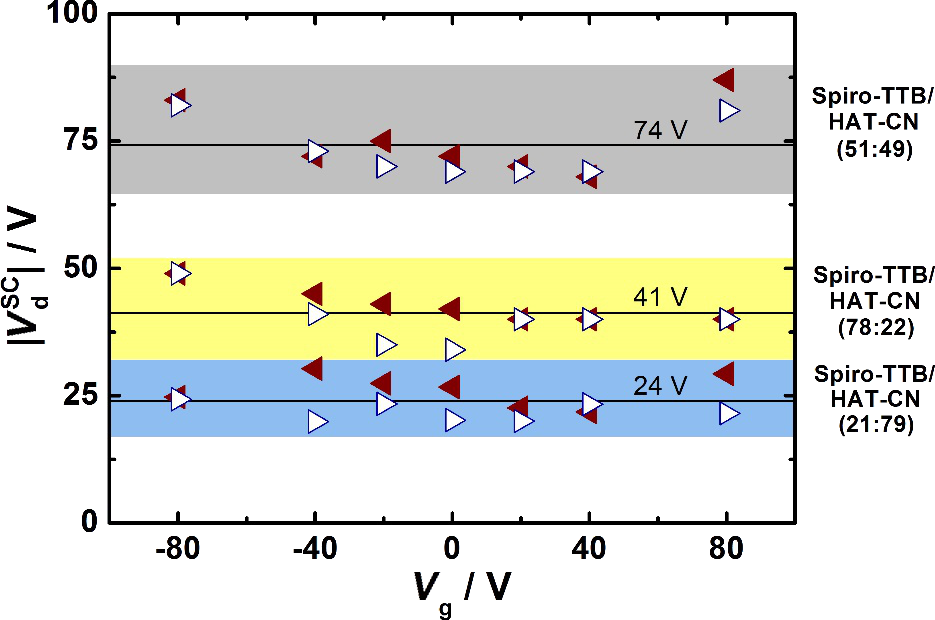
The drain voltage *V*_d_^SC^, at which the sign reversal takes place, is plotted for different *V*_g_ for all mixing ratios. All points belonging to one mixing ratio are highlighted in a separate colour. Red triangles denote the measurements with positive *V*_d_, while white triangles represent negative *V*_d_. The solid black lines represent the average *V*_d_^SC^ values for each mixing ratio.

We define *V*_d_^SC^ as the drain voltage at which the magnetoresistance sign-change takes place. In order to estimate *V*_d_^SC^ the MR(*V*_d_)-curves at different values of *V*_g_ were fitted with a simple exponential function. The resulting values of *V*_d_^SC^ are summarized in Figure 3 as a function of *V*_g_ for the different mixing ratios. The sign reversal takes place at 74 V for a mixing ratio of 51:49. The *V*_d_^SC^ values for the mixing ratios of 78:22 and 21:79 are significantly lower with values of 41 V and 24 V, respectively. Based on these results we can conclude that the MR landscape as well as the sign of the magnetoresistance can be electrically controlled in transistor structures based on Spiro-TTB/HAT-CN films. Furthermore, the corresponding *V*_d_^SC^ values of the voltage-induced sign reversal can be significantly influenced by the composition of the donor/acceptor system.

**Figure 4:**
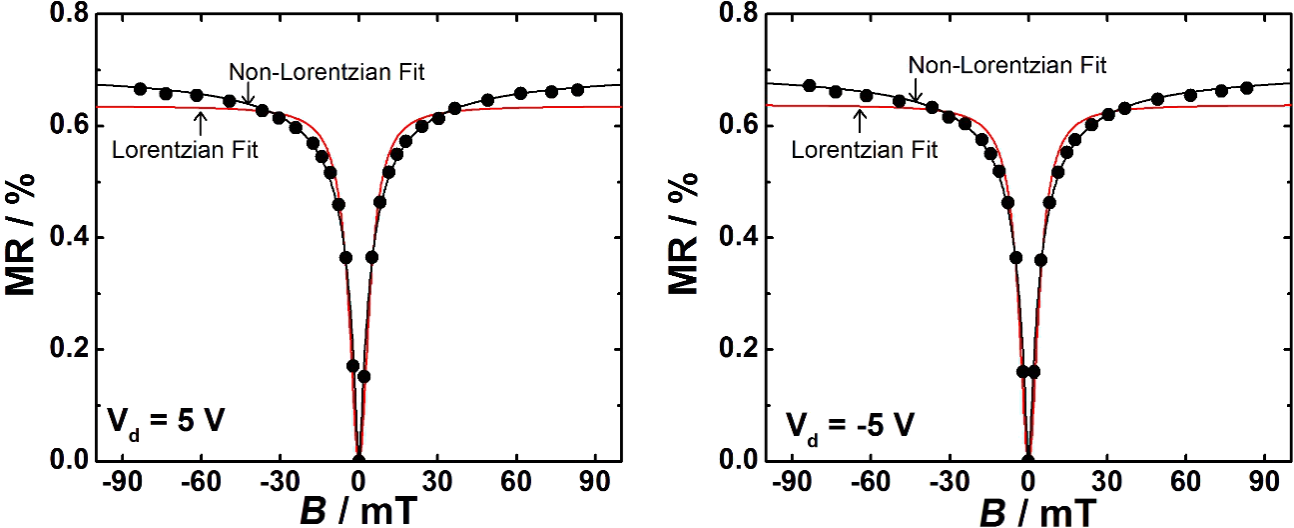
Representative MR line shape curves are shown at *V*_d_ = −5 V and +5 V. Black and red lines indicate fits with a Lorentzian (Supporting Information File 1, Equation S1) and a non-Lorentzian function (Supporting Information File 1, Equation S2), respectively. Hereby, devices with a mixing ratio of 51:49 were used. The applied magnetic fields are higher than 2 mT and *V*_g_ was zero for all measurements.

**Figure 5:**
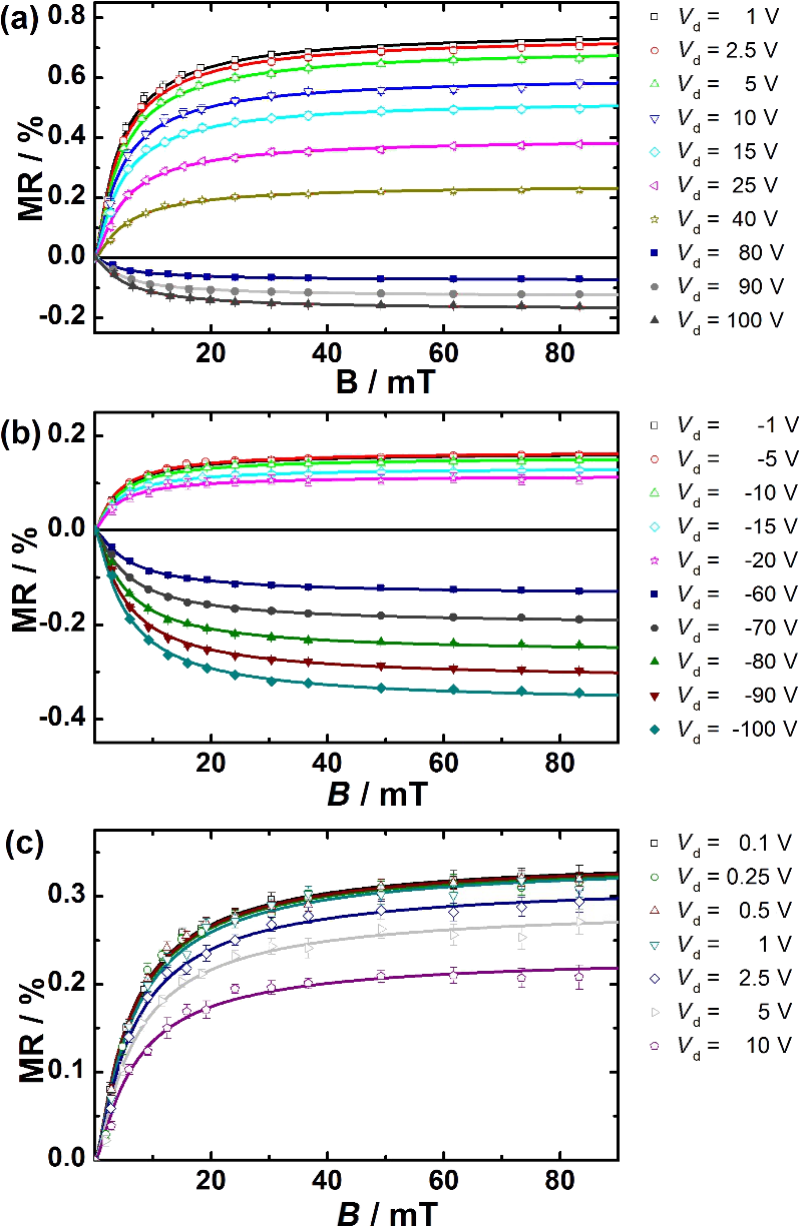
The dependence of the MR line shape curves on the drain voltage *V*_d_ is shown for a mixing ratio of (a) 51:49, (b) 78:22 and (c) 21:79. All measurements were performed at *V*_g_ fixed at zero voltage.

For a detailed analysis of the MR curves, separate measurement series were performed for small fields (2 mT < *B* < 85 mT) and ultrasmall fields (0.5 mT < *B* < 5 mT). First, a typical MR line shape curve for magnetic fields between 2 and 85 mT is displayed in Figure 4. It becomes clear that the non-Lorentzian line shape fits our data better than the Lorentzian line shape. This holds true for all Spiro-TTB/HAT-CN compositions (See also Table S1, S2 and S3 in Supporting Information File 1). Figure 5 shows the MR line shapes for all mixing ratios obtained at different *V*_d_. Increasing *V*_d_ results in a reduction of the positive magnetoresistance in all mixing ratios and a clear magnetoresistance sign-change can be tailored for mixing ratios of 51:49 and 78:22.

**Figure 6:**
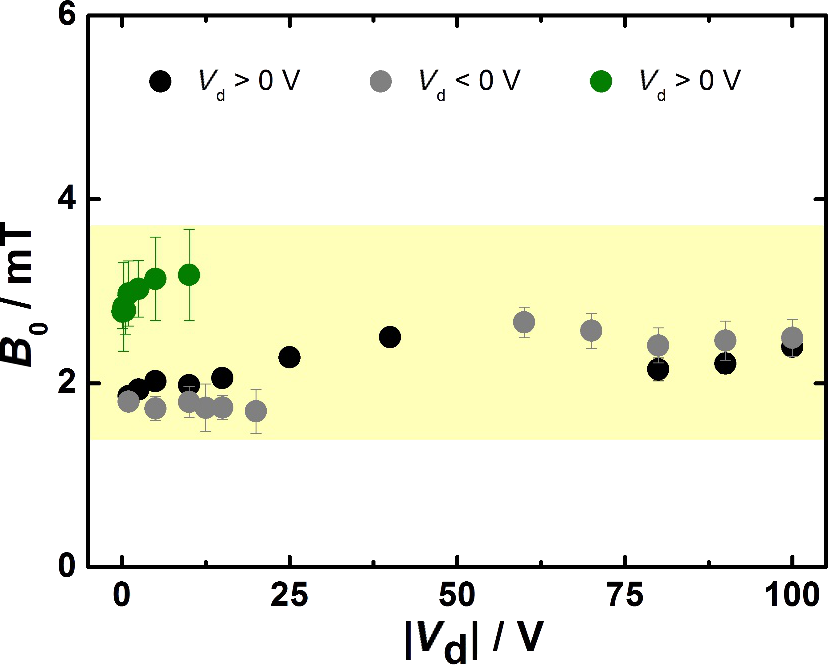
Voltage dependence of the line shape width *B*_0_. The values of *B*_0_ were obtained from fitting the data with the non-Lorentzian fit function.

Derived from the fitting results presented in Figure 5 we obtained *B*_0_ values for all measurements as displayed in Figure 6. The values of *B*_0_ for all compositions are quite similar in magnitude and relatively independent of the applied voltage. Thus, the average *B*_0_ value is estimated to be 2.11 ± 0.06 mT, which can be regarded as representative value for all coevaporated Spiro-TTB/HAT-CN compositions. The fit parameter *B*_0_ is often correlated with the strength of the molecular hyperfine fields that affect the magnetosensitive quasiparticles [7,11,20–21]. Furthermore, it depends on the microscopic details of the underlying model [22]. The voltage and composition independence of *B*_0_ suggests that one specific quasiparticle type can explain the entire magnetoresistive behaviour. Therefore, both the positive and the negative magnetoresistance should derive from one specific elementary process and the sign reversal should not be based on different components.

**Figure 7:**
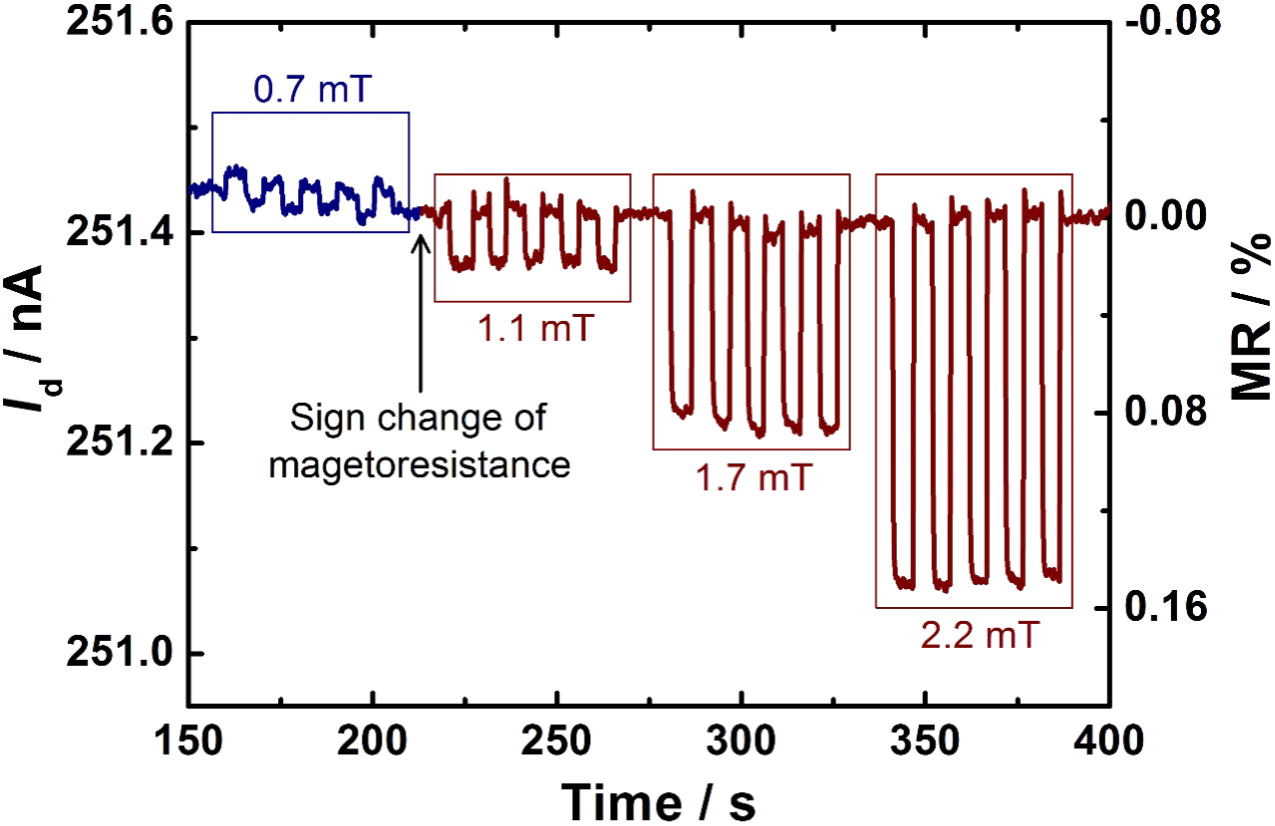
Experimental raw data covering ultrasmall magnetic-field effects including a MR sign-reversal. For *B* = 0.7 mT the *I*_d_ is increased (highlighted in blue) while *I*_d_ decreases for larger magnetic fields (highlighted in red). The experiment was carried out for zero *V*_g_ = 0, *V*_d_ = −2.5 V and a mixing ratio of 51:49.

Furthermore, our experimental data shows a significant and reproducible influence of ultrasmall magnetic fields on *I*_d_ for *B* < 5 mT including an additional magnetic field induced MR sign-change (Figure 7). At a magnetic field of ≈1 mT, the effect of ultrasmall fields is reversed, resulting in a different MR-sign for *B* < 1 mT, than for *B* > 1 mT is obtained. So far, experimental evidence for this phenomenon, which is known as the ultrasmall magnetic field effect (USMFE), has only been provided by two research groups in organic diodes [19,23–26]. Here, we are able to verify it for the first time in organic transistors.

Up to now, USMF effects have been discussed controversially in the scientific community. For example, they have been explained based on the energetic crossover (and thus the increase of the spin mixture) of spin sublevels of correlated polaron pairs, coupled to nuclear spins [20,24,26]. Alternatively, the competition between the exciton or bipolaron formation and spin mixing serves as an explanation for USMF effects [22,27–29]. Both explanatory approaches have in common that they are applicable to neutral and charged spin pairs and the quasi-particles can be bipolarons, excitons or electron hole pairs. USMFE could be detected in both unipolar and bipolar diodes and they represent a fundamental component of the MR line shape [24–26]. Herby, simple non-Lorentzian and Lorentzian functions are not suitable to fit MR line shapes including the more complex USMF effects. Instead, a theory developed by Bobbert and co-workers has been used to fit USMFE according to the following equation [22]:

[1]
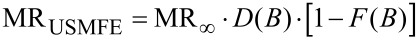


MR_∞_ is the maximum magnetoresistance, which can be achieved in the "slow hopping regime" at infinitely high magnetic-field strength. *D*(*B*) is a dephasing factor to account for the average differences in the precession frequency of interacting polaron spins. *F*(*B*) is the form factor, allowing reproducing both, the non-Lorentzian and Lorentzian line shapes. This factor includes model specific components responsible for line shape broadening, which does not derive from the strength of the hyperfine fields. In the following, MR_USMFE_(*B*) is used to illustrate the MR line shape together with the USMF effects.

**Figure 8:**
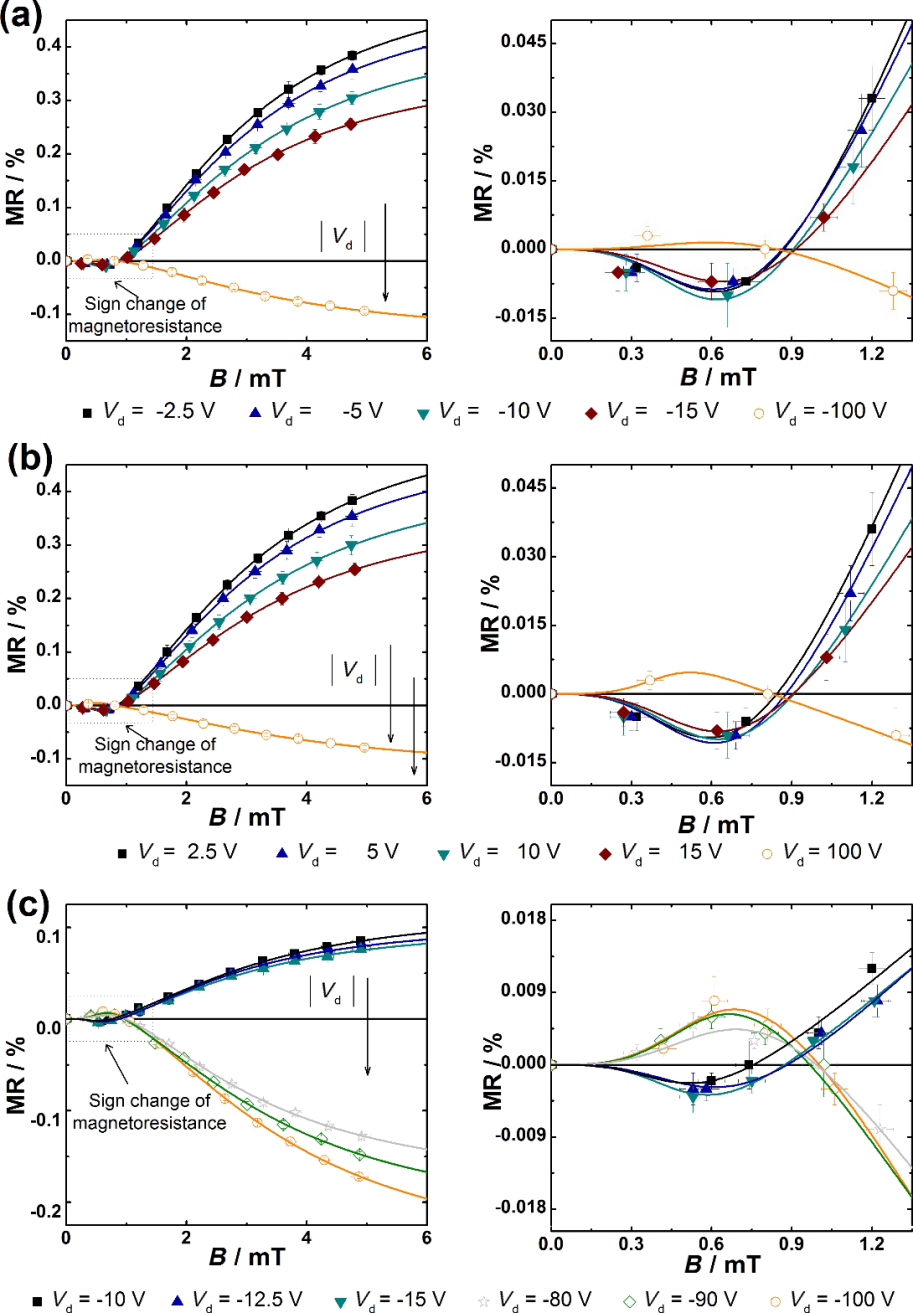
Ultrasmall magnetic field effects obtained for different compositions of Spiro-TTB/HAT-CN systems with a mixing ratio of (a) and (b) 51:49 and (c) 78:22 [19]. The measurements were performed at different values of *V*_d_ while keeping *V*_g_ constant at zero voltage. The solid lines are the fitting curves according to Equation 1.

USMFE could be detected in transistor structures with different mixing ratios of Spiro-TTB/HAT-CN and are presented as a function of *V*_d_. The corresponding results, including the fits based on the function MR_USMFE_ (Equation 1), are displayed in Figure 8. They show that a MR sign-change takes place in all measurements at a magnetic field strength of ≈1 mT. For low to moderate *V*_d_ the sign of the MR changes from negative (*B* < 1 mT) to positive (*B* > 1 mT). This behavior is reversed for higher *V*_d_, where MR is changing from positive (*B* < 1 mT) to negative (*B* > 1 mT). Our results are quite similar as obtained for π-conjugated polymers [20,23,26]. The USMFE in coevaporated Spiro-TTB/HAT-CN systems is sensitive to the voltage conditions and *V*_d_ can control the sign of MR. This dependence on *V*_d_ is analogous to the effects at moderate magnetic field strengths (5 mT < *B* < 85 mT) and the USMFE is subject to the same trends (see Figure 2 and Figure 5).

**Figure 9:**
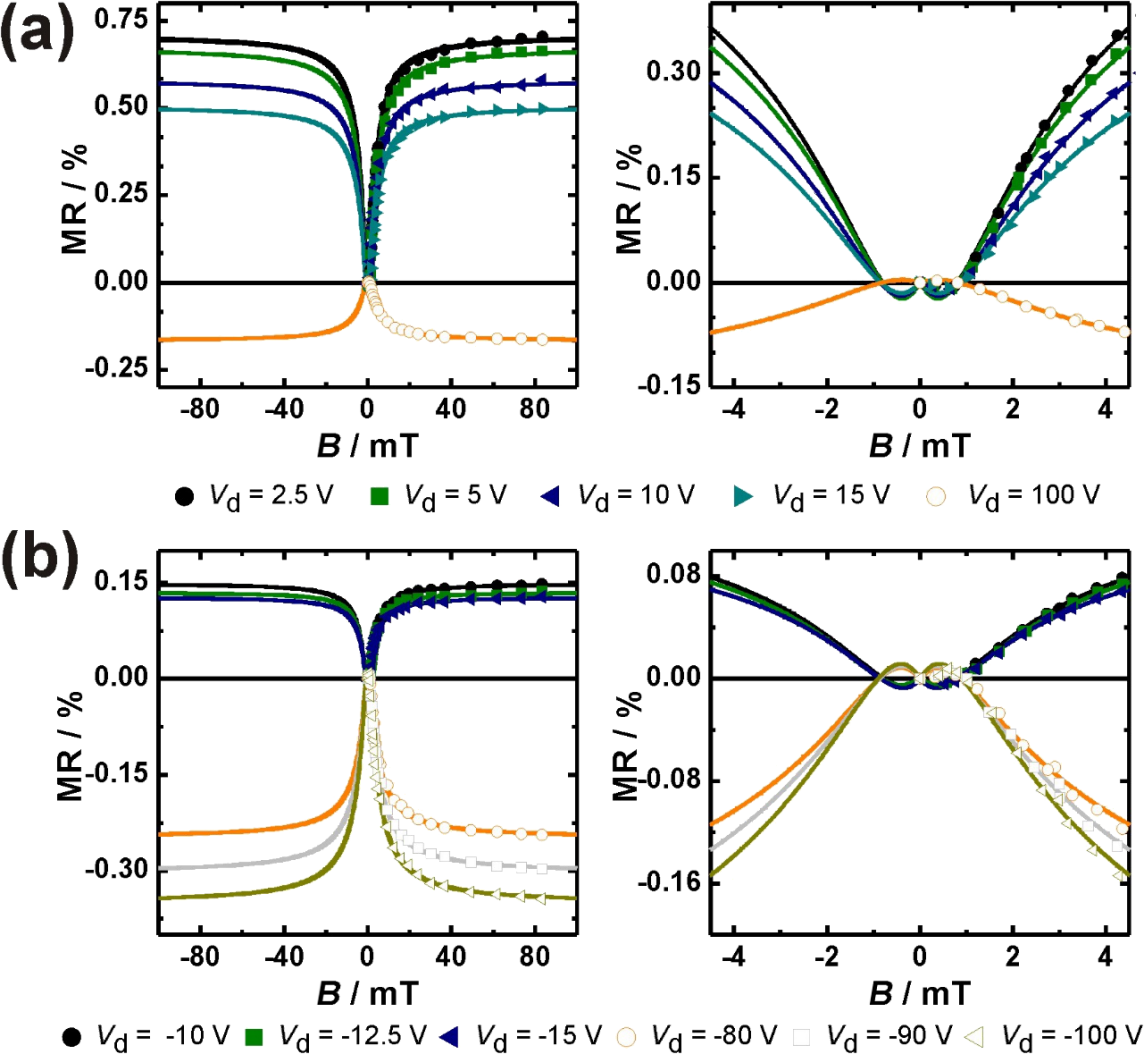
Magnetic-field effects in transistors based on different composition of Spiro-TTB and HAT-CN with a mixed ratio of (a) 51:49 and (b) 78:22 measured at different values of *V*_d_ are shown. The high quality of fit in the saturation regime can be seen in the left side. The right side gives evidence of sign reversal of magnetoresistance and the ultrasmall magnetic field effect. The solid lines are fitting curves according to Equation 1. All measurements were carried out at zero gate voltage.

Now we combine the experimental data obtained from small fields (2 mT < *B* < 85 mT) and ultrasmall fields (0.5 mT < *B* < 5 mT). The fit function of MR_USMFE_ (Equation 1) is used to illustrate the curve shape over the complete measuring range (0.5 mT < *B* < 85 mT). The combined measurement results are shown in Figure 9 together with the fits based on MR_USMFE_ [19]. We have shown that both the non-Lorentzian line shape for small magnetic field strengths as well as the magnetic field induced MR sign change at ultrasmall magnetic field strengths are successfully fitted for all measured *V*_d_. The saturation behaviour of the line shape and USMFE can be described simultaneously and the fit function MR_USMFE_ proves to be suitable for the complete MR values. The results of the separate measurement series are thus integrated into a uniform functional context.

Our results show for the first time ultrasmall magnetic-field effects in organic transistors. Under moderate drain voltage conditions; there is a positive magnetoresistance, which changes its sign at large drain voltages. Independent of the mixing ratio as well as the applied drain or source voltages, similar non-Lorentzian like MR line shapes and an average *B*_0_ value of ≈2.1 mT are observed. The individual line shape was recently used as the most important criterion for the identification of the underlying magnetosensitive process [25,30]. Since all curves result in similar *B*_0_-values, one can conclude that the magnetoresistance effects in Spiro-TTB/HAT-CN compositions depend on one specific quasiparticle species and the corresponding model should be capable to explain both positive and negative magnetoresistance. The low *B*_0_ value of ≈2.1 mT is a typical characteristic for low-fields effects based on spin mixing due to the molecular hyperfine fields [22,25,30]. In principle, low-fields effects can be achieved with the electron–hole pair, exciton–polaron interaction and the bipolaron model [25,30–31]. In the absence of specific assumptions, only the unmodified bipolaron model leads to positive magnetoresistance. In addition, other low-field concepts appear to be less likely to explain all magnetoresistance effects in coevaporated Spiro-TTB/HAT-CN systems, alternative explanations cannot be excluded. In particular magnetic-field sensitive intermolecular radical pair states [18] as well as a magnetic-field dependent electron transfer between donor–acceptor units may also play a role [32]. However, our experimental findings can be satisfactorily elucidated within the framework of the bipolaron model.

Bipolarons can be stabilized by the presence of counter charges [33–35]. Furthermore, the coulomb repulsive interaction occurring in the formation of bipolarons species can be compensated by a correspondingly high energetic disorder of the thin films [11,22,36–38]. Doping of amorphous organic films results in a broadening of the density of states and, thus, is increasing the energetic disorder [39–40]. Therefore, the high density of intrinsic charges in Spiro-TTB/HAT-CN compositions as well as the broadened density of states leads to an increased probability for the formation of bipolaronic species. According to the bipolaron model, two equally charged polarons can only simultaneously occupy a molecular transport site if their spin states are antiparallel [11,37]. For parallel polaron spins, bipolaron formation is energetically forbidden. Thus, the formation of bipolarons depends on the relative orientation of the precursor polaron spins and the process is magnetosensitive. The singlet and triplet states of the precursor polarons pairs are energetically degenerated in the absence of external magnetic fields and are mixed by the statistically distributed hyperfine fields. In magnetic fields larger than the hyperfine fields, the Zeeman splitting decreases the energetic degeneracy and the spin mixture is reduced [11,37]. Since the spin mixture enhances the generation of (singlet) bipolarons, the magnetic field decreases the probability of bipolaron formation. Typically, more free charge carriers are blocked. This results in a decrease in mobility and we obtain positive magnetoresistance. On the other hand, if a large number of bipolarons is present, the effect of the magnetic field is reversed [11,22]. At a certain number of bipolarons no sufficient number of free charge carriers is available and the charge transport is hindered. In this scenario, the magnetic field induced reduction of the spin mixture and thus, the reduced bipolaron formation-probability leads to a release of charge carriers, resulting in an increase of the current. Finally, negative magnetoresistance is obtained. In the context of the bipolaron model, the magnetoresistive behaviour of Spiro-TTB/HAT-CN compositions can be discussed as follows: The doping process between Spiro-TTB and HAT-CN in thin films generates intrinsic counter charges and increases the energetic disorder, which favours the formation of bipolarons. The formation process is spin-sensitive and the number of bipolarons is reduced for an increasing external magnetic field. At low drain voltage, a moderate number of bipolaron species is present and positive magnetoresistance is obtained. For larger *V*_d_ values, the current density increases and, thus, the probability of individual polarons to meet each other increases as well. Hence, more bipolarons are formed at high drain voltages and negative magnetoresistance gets predominant. Since positive and negative magnetoresistance are derived from the same quasiparticle species, whose magnetosensitivity is based on the spin mixture by hyperfine fields, ultrasmall magnetic-field effects are observed. The USMFE can also be described within the context of the bipolaron model. In unipolar diodes, the effect can be attributed to the change in singlet-triplet mixing near the energetic crossover of spin sublevels of bipolaronic species coupled to nuclear spins [23,25]. Alternatively, the competition between the bipolaron formation and the spin mixing is used to explain USMFE [27,29]. Both concepts propose an initial increase of spin mixing at ultrasmall magnetic fields. For stronger magnetic fields, the reduction of the spin mixing due to the Zeeman decoupling of the singlet and triplet levels dominates leading to the MR sign-change typical for USMFE. The polarity of the MR sign-change is controlled by the drain voltage, which can be explained with the previously discussed arguments.

## Conclusion

Magnetoresistance in organic transistors has been proven for the first time without additional illumination by employing a mixed system of Spiro-TTB/HAT-CN. Intermolecular charge transfer between both molecules results in high intrinsic charge carrier density in the mixed films, which is sensitive to magnetic fields. The current flow can also be efficiently modulated with ultrasmall magnetic fields. A magnetic field-induced MR sign change occurs at *B* ≈ 1 mT (USMFE). The magneto-resistive behavior is sensitive to the voltage conditions and the MR sign can be reversed by the drain voltage. The magnetoresistance effects can be successfully described within the framework of the bipolaron model, whereby the formation of the magnetosensitive bipolaronic species is made possible by the presence of (counter) charges present in the Spiro-TTB/HAT-CN mixed system. The ionizing donor–acceptor interaction can thus be regarded as a promising concept for the generation of highly conductive, magnetosensitive transport layers. Hence, organic transistors appear to represent an upcoming platform for studying spin-dependent processes in molecular semiconductors thereby leading the way towards efficient, multifunctional organic spin-devices.

## Experimental

Bottom-contact field-effect transistor substrates were purchased from Fraunhofer IPMS (Dresden, Germany) with channel lengths (*L*) between 2.5 and 20 µm and channel width (*W*) of 10 mm. The isolation layer consists of 230 ± 10 nm thick SiO_2_ and the source and drain electrodes are 30 nm Au with 10 nm ITO as adhesion layer. For all experiments transistors were used with *L* = 2.5 µm and *W* = 10 mm which are measured in a glove box (O_2_, H_2_O < 0.1 ppm) at room temperature. Spiro-TTB and HAT-CN were synthesized and purified in our laboratory. Prior to the deposition of Spiro-TTB and HAT-CN, the predefined substrates were cleaned with acetone, 2-propanol and deionized water, followed by oxygen-plasma treatment and exposure to hexamethyldisilazane to replace the natural hydroxyl end group of SiO_2_ with an apolar methoxy group. Finally, Spiro-TTB and HAT-CN were deposited by thermal evaporation at a base pressure of 1 × 10^−7^ Torr (*T*_substrate_ = 298 K) with a thickness of 40 nm. For mixed system, the composition was controlled by using different evaporation rates for Spiro-TTB and HAT-CN, respectively. The mixing ratios (mass ratios) between Spiro-TTB and HAT-CN are 21:79, 51:49 and 78:22, respectively. The evaporation rates were monitored by two independent oscillating quartz-sensors. The uncertainty of our deposition process is ±2.5%. From the vacuum chamber, the samples were directly transferred to a glove box (O_2_, H_2_O < 0.1 ppm) and placed in a homebuilt sample holder. This sample holder was placed between the poles of an (unshielded) electromagnet with the magnetic field being perpendicular to the direction of the current flow in OFETs. The magnetic field was varied between −85 mT and +85 mT. Due to the (unshielded) earth magnetic field, reminiscence effects of the electromagnet and the uncertainty of our magnetic-field sensor, the uncertainty of the stated magnetic-field strength is ±80 µT. Current–voltage measurements were performed by using a Keithley 4200 semiconductor characterization system equipped with preamplifiers for improving low-current measurements. All measurements were performed at room temperature (≈298 K).

## Supporting Information

File 1 The dependency of magnetoresistance on the drain and gate voltage for Spiro-TTB/HAT-CN for different mixing ratios and resulting fit parameters.
