# Supplementary material for: Ultrasmall magnetic field-effect and sign reversal in transistors based on donor/acceptor systems
Source: Beilstein J Nanotechnol. 2017 May 19;8:1104–14. doi: 10.3762/bjnano.8.112 (PMC5480331; doi:10.3762/bjnano.8.112)
Supplement: File 1 — The dependency of magnetoresistance on the drain and gate voltage for Spiro-TTB/HAT-CN for different mixing ratios and resulting fit parameters. [file Beilstein_J_Nanotechnol-08-1104-s001.pdf]

Supporting Information

for

Ultrasmall magnetic field-effect and sign reversal in transistors based on donor/acceptor systems

Thomas Reichert[1] and Tobat P. I. Saragi*[1,2]

Address: [1]Macromolecular Chemistry and Molecular Materials, Department of Mathematics and Science, Center for Interdisciplinary Nanostructure Science and Technology (CINSaT), University of Kassel, Heinrich-Plett-Straße 40, 34132 Kassel, Germany and [2]Present address: Accenture GmbH, Kaistraße 20, 40221 Düsseldorf, Germany

Email: Tobat P. I. Saragi* - tobat.saragi@gmx.de

* Corresponding author

The dependency of magnetoresistance on the drain and gate voltage for Spiro-TTB/HAT-CN for different mixing ratios and resulting fit parameters

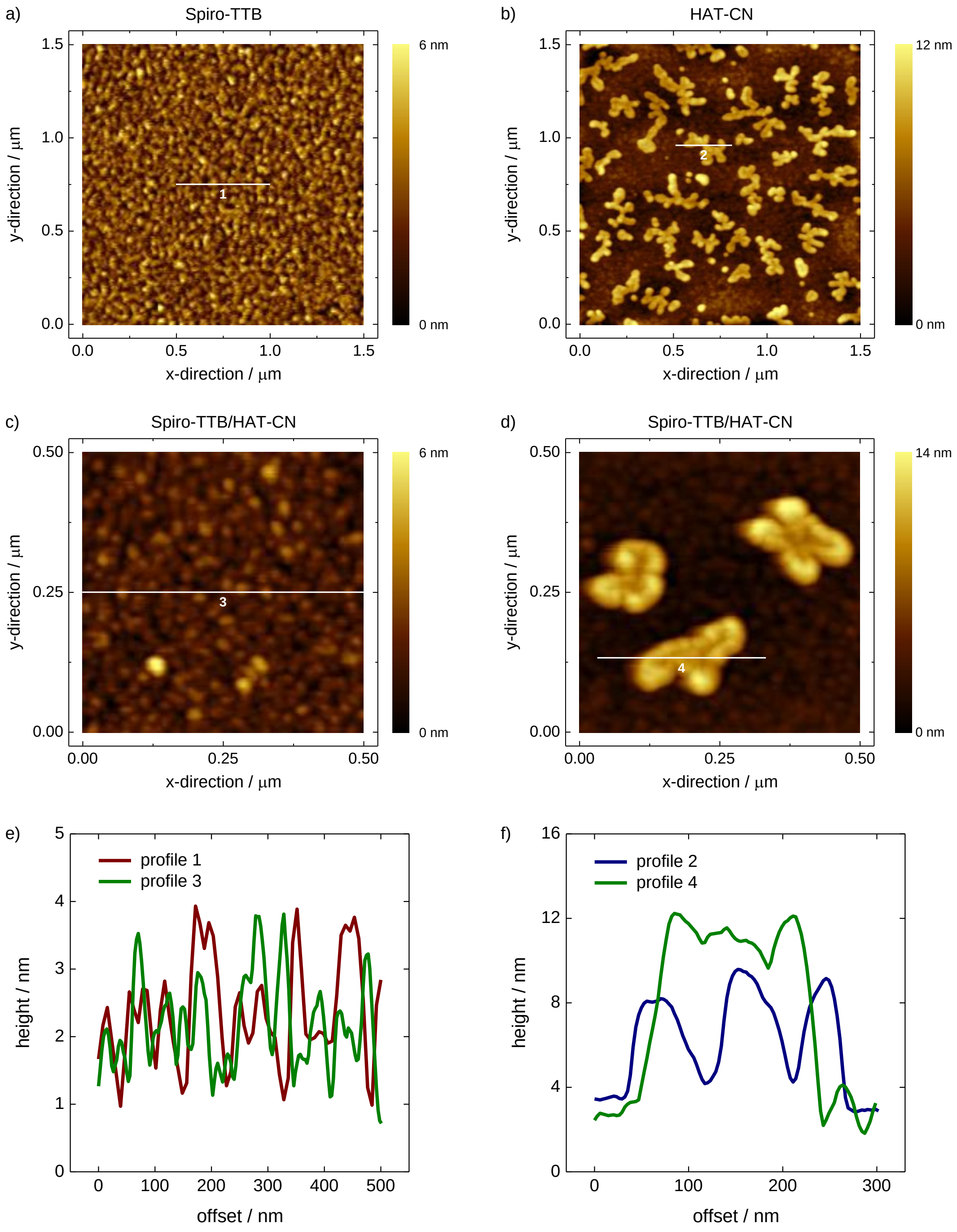

Figure S1: AFM measurements of a) 130 nm thin Spiro-TTB films, b) 1 nm thin HAT-CN films and c), d) 20 nm thin coevaporated Spiro-TTB/HAT-CN films (51:49) as well as e), f) representative height profile-lines of the corresponding surface structures are shown. The mixed Spiro-TTB/HAT-CN thin-film system contains relative smooth areas with similar surface features as individual Spiro-TTB films *and* island-like structures of similar form and dimension as the surface structures of individual HAT-CN films.

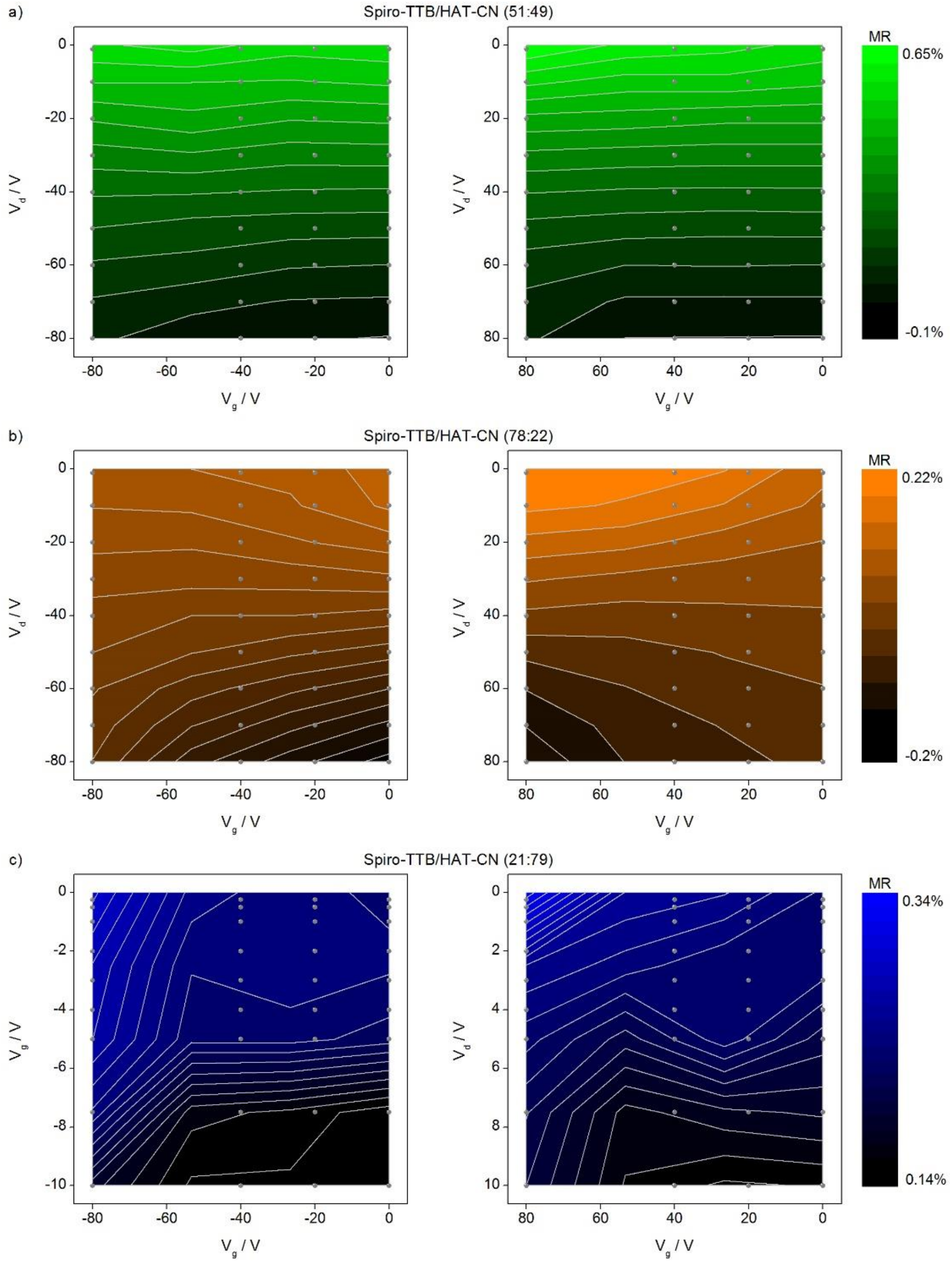

Figure S2: The dependency of magnetoresistance on the drain and gate voltage for Spiro-TTB/HAT-CN for different mixing ratios: (a) (51:49), (b) (78:22) and (.c) (21:79). Graphs on the left and right side refer to *p*-channel and *n*-channel conditions, respectively. Values between the experimental data was interpolated with the *Renka-Cline Fit*. Experimental data-points are highlighted by grey dots. A colour scaling of the MR values is integrated as well. It applies to both graphs of one mixing ratio. All measurements were carried out for *B* = 60 mT.

Table S1: Fit results of the MR(B) curves for Spiro-TTB/HAT-CN with a mixing ratio of 51:49 at different drain voltages.

V_d [V]	Non-Lorentz Fit			Lorentz Fit		
	MR_∞^a [mT]	B_0^a [mT]	QF^b	MR_∞^a [mT]	B_0^a [mT]	QF^b
	Spiro-TTB/HAT-CN (1:1)					
1	0.760±0.015	1.86±0.09	0.992	0.692±0.020	4.32±0.56	0.944
2.5	0.743±0.017	1.93±0.10	0.991	0.676±0.018	4.44±0.50	0.950
5	0.701±0.008	2.02±0.08	0.992	0.636±0.013	4.51±0.40	0.960
-5	0.703±0.008	1.95±0.10	0.990	0.638±0.013	4.45±0.39	0.961
10	0.608±0.010	1.98±0.08	0.993	0.552±0.017	4.62±0.59	0.941
15	0.529±0.010	2.06±0.08	0.992	0.479±0.015	4.74±0.61	0.940
25	0.401±0.007	2.28±0.09	0.992	0.361±0.012	5.18±0.67	0.933
40	0.244±0.005	2.50±0.10	0.990	0.219±0.006	5.66±0.60	0.940
80	-0.076±0.002	2.15±0.12	0.988	-0.069±0.002	4.96±0.66	0.934
90	-0.131±0.003	2.22±0.11	0.990	-0.118±0.003	5.16±0.50	0.950
100	-0.175±0.004	2.40±0.10	0.990	-0.158±0.004	5.54±0.58	0.941

[a]Applied fit parameters according to Equations S1 and S2. The Lorentzian line shape can be described by the following equation:

$$MR(B) = MR_\infty \frac{B^2}{B^2 + B_{0'}^2} \qquad \text{(S1)}$$

while the Non-Lorentzian line shape is written as

$$MR(B) = MR_\infty \frac{B^2}{(|B| + B_0)^2} \qquad \text{(S2)}$$

whereas B is the applied magnetic field, MR_∞ is the saturation value of MR for an infinite magnetic-field strength, $B_{0'}$ gives the half width at half maximum of MR and B_0 gives the half width at quarter maximum of MR.

[b]QF = Quality of fit. The best fit has a QF value of 1.

Table S2: Fit results of the MR(B) curves for Spiro-TTB/HAT-CN with a mixing ratio of 78:22 at different drain voltages.

V_d [V]	Non-Lorentz Fit			Lorentz Fit		
	MR_{∞}^{a} [mT]	B_0^{a} [mT]	QF^{b}	MR_{∞}^{a} [mT]	B_0^{a} [mT]	QF^{b}
	Spiro-TTB/HAT-CN (4:1)					
-1	0.166±0.001	1.80±0.07	0.993	0.151±0.005	4.28±0.67	0.933
-5	0.168±0.002	1.73±0.13	0.987	0.154±0.005	4.17±0.58	0.942
-10	0.156±0.003	1.80±0.17	0.983	0.143±0.004	4.32±0.48	0.952
-12.5	0.143±0.004	1.73±0.26	0.974	0.130±0.003	4.06±0.41	0.960
-15	0.133±0.002	1.74±0.13	0.987	0.122±0.004	4.18±0.53	0.947
-20	0.116±0.003	1.70±0.24	0.976	0.107±0.003	4.18±0.46	0.954
-60	-0.138±0.002	2.66±0.17	0.983	-0.123±0.005	5.94±0.81	0.919
-70	-0.201±0.004	2.57±0.19	0.981	-0.178±0.006	5.63±0.70	0.930
-80	-0.262±0.005	2.41±0.19	0.981	-0.235±0.003	5.49±0.66	0.934
-90	-0.319±0.006	2.46±0.21	0.979	-0.286±0.008	5.57±0.62	0.938
-100	-0.369±0.007	2.49±0.20	0.980	-0.369±0.007	5.64±0.65	0.935

[a]Applied fit parameters according to Equations S1 and S2.

[b]QF = Quality of fit. The best fit has a QF value of 1.

Table S3: Fit results of the MR(B) curves for Spiro-TTB/HAT-CN with a mixing ratio of 21:79 at different drain voltages.

V_d [V]	Non-Lorentz Fit			Lorentz Fit		
	MR_∞[a] [mT]	B_0[a] [mT]	*QF*[b]	MR_∞[a] [mT]	B_0[a] [mT]	*QF*[b]
	Spiro-TTB/HAT-CN (1:4)					
0.1	0.348±0.005	2.78±0.18	0.982	0.309±0.012	6.16±0.88	0.911
0.25	0.344±0.014	2.83±0.48	0.952	0.305±0.009	6.12±0.69	0.931
0.5	0.345±0.008	2.79±0.26	0.974	0.308±0.010	6.18±0.74	0.926
1	0.342±0.010	2.98±0.35	0.965	0.303±0.011	6.52±0.84	0.916
2.5	0.318±0.008	3.03±0.31	0.969	0.281±0.011	6.54±0.89	0.911
5	0.289±0.011	3.13±0.45	0.955	0.256±0.009	6.68±0.82	0.918
10	0.234±0.010	3.18±0.50	0.950	0.207±0.007	6.93±0.77	0.923

[a]Applied fit parameters according to Equations S1 and S2.

[b]QF = Quality of fit. The best fit has a QF value of 1.
